# Efficacy and Safety of Thalidomide As a Pre-Medication of Chemotherapy-Induced Nausea and Vomiting (CINV) Following Highly Emetogenic Chemotherapy (HEC): A Systematic Review and Meta-Analysis

**DOI:** 10.3389/fonc.2021.818839

**Published:** 2022-01-24

**Authors:** Jiyi Xie, Cong Zhang, Shijun Li, Rong Dai, Mitchell A. Sullivan, Bin Deng, Qiling Xu, Jinglin Wang, Chen Shi, Yu Zhang

**Affiliations:** ^1^ Department of Pharmacy, Union Hospital, Tongji Medical College, Huazhong University of Science and Technology, Wuhan, China; ^2^ Hubei Province Clinical Research Center for Precision Medicine for Critical Illness, Wuhan, China; ^3^ Glycation and Diabetes Group, Mater Research Institute - The University of Queensland, Translational Research Institute, Brisbane, QLD, Australia

**Keywords:** chemotherapy-induced nausea and vomiting, thalidomide, safety, efficacy, highly emetogenic chemotherapy

## Abstract

**Background:**

In China, thalidomide (THD) has been used to prevent chemotherapy-induced nausea and vomiting (CINV) following highly emetogenic chemotherapy (HEC); however, there is limited evidence on the efficacy and safety of THD in this setting. The aim of this study was to evaluate the efficacy, safety, and impact on quality of life (QoL) of THD on CINV following HEC.

**Methods:**

Electronic databases were systematically searched for all randomized controlled trials (RCTs) in HEC using THD. The primary outcomes were complete response (CR) and no nausea, Secondary outcomes were the incidence of adverse events and QoL related indicators. We calculated risk ratios (RRs) and 95% confidence intervals (CIs) using a fixed-effects model. In the case of heterogeneity (I^2^≥50%), a random-effects model was performed.

**Results:**

A total of 3168 patients were included from 34 RCTs. In terms of CR rate, THD plus 5-HT_3_ receptor antagonist (5-HT_3_RA) with or without dexamethasone (DEX) was significantly higher than 5-HT3RA with or without DEX in the acute phase (74.4% vs 67.4%; RR 1.10), delayed phase (70.6% vs 50.4%; RR 1.53), and overall phase (68.4% vs 53.4%; RR 1.28). In terms of no nausea rate, the THD group was also significantly higher than the control group in the acute phase (61.7% vs 55.5%; RR 1.12), delayed phase (50.5% vs 30.0%; RR 1.69), and overall phase (44.6% vs 29.9%; RR 1.50). There was no statistical difference in the incidence of fatigue, headache, diarrhea, rash, hepatorenal damage, and myelosuppression between those with and without THD. The incidence of increase in KPS scores, weight gain, appetite improvement, and sleep quality improvement were significantly higher with the addition of THD.

**Conclusions:**

THD may be effective and safe for the prevention of CINV patients treated with HEC and may improve QoL.

## 1 Introduction

Chemotherapy-induced nausea and vomiting (CINV) is one of the most common disturbing adverse effects of anticancer chemotherapy, which can significantly impair the patient’s quality of life (QoL), adherence with future therapy, and nutritional status. American Society of Clinical Oncology (ASCO) guideline (2020) (1) classify chemotherapeutic agents according to their emetogenic potential (high, medium, low and minimal) and make recommendations based on their level of risk. For patients receiving highly emetogenic chemotherapy (HEC; CINV risk>90%), such as cisplatin- and anthracycline/cyclophosphamide (AC)-based regimens, National Comprehensive Cancer Network (NCCN) antiemesis guideline recommend a four-drug combination of a 5-HT_3_ receptor antagonist (5-HT_3_RA), a neurokinin-1 (NK1) RA, dexamethasone (DEX), and olanzapine (2). Even if CINV prevention is now dramatically improved, there is still a need to find more effective, safer and more economical drug regimens for better prevention because CINV remains a frequent and feared adverse effect.

The unintended teratogenic effect of thalidomide (THD), prescribed to treat morning sickness in pregnant women, is a historic tragedy, however with the approval of this drug for indications such as multiple myeloma. A randomized controlled double-blind phase III clinical study (3) in the Chinese population suggested that THD combined with palonosetron and DEX is efficacious and well-tolerated for the prevention of delayed CINV in anticancer chemotherapy-naive patients who undergo HEC. Rates of complete response and no nausea in the delayed phase were higher and adverse effects were mild to moderate in the THD group. Since pregnancy and childbirth are nearly impossible during anticancer chemotherapy in patients with malignant tumors, and THD prices are relatively low in China, there is some potential for THD to be useful in the management of CINV.

In China, there have been many controlled clinical trials using THD, in addition with antiemetic regimens, with results showing that THD can be used as a complementary and alternative medicine to prevent CINV following HEC. However, there is no systematic review or meta-analysis of its efficacy in the prevention of CINV, the incidence of adverse effects, and the improvement of QoL under HEC. Therefore, all controlled clinical trials using THD under HEC were systematically evaluated for efficacy in the prevention of CINV through multiple studies and large sample size.

## 2 Methods

The meta-analysis was pre-registered at PROSPERO (CRD42020158732).

### 2.1 Literature Search

This systematic review and meta-analysis was conducted and reported according to the Preferred Reporting Items for Systematic Reviews and Meta-analyses (PRISMA) guidelines (4). Relevant publications were searched in the Chinese National Knowledge Infrastructure (CNKI), the VIP Information Database, Wanfang Database, PubMed, EMBASE, and the Cochrane Library. The systematic review was performed in December 2019 and updated in August 2020.

The keywords for searching included: “chemotherapy-induced nausea and vomiting”, “CINV”, “vomit”, “emesis”, “thalidomide”, “highly emetogenic chemotherapy”, “CDDP”, “cisplatin”, or “anthracycline and cyclophosphamide”. References of the selected articles were also checked to identify further eligible trials.

### 2.2 Study Selection Criteria

Selecting studies that met the inclusion and exclusion criteria was independently performed by two authors(JX, CZ). Any disagreement between reviewers was resolved through public discussions until a consensus was reached.

Inclusion criteria: (a) randomized controlled trials (RCTs) in patients who received HEC (such as cisplatin-based treatment or AC regimen); (b) studies that reported either THD as an add-on treatment (5-HT_2_RA, with or without DEX) or THD monotherapy compared to standard treatment.

Exclusion criteria: (a) review articles or studies involving non-human subjects; (b) duplicate published articles; (c) studies where anticancer chemotherapy regimens and basic antiemetic regimens were inconsistent between experimental and control groups; (d) studies with a high risk of bias.

### 2.3 Outcomes

The primary outcomes: Complete response (CR) and no nausea. CR is defined as having no emetic episode and requiring no use of rescue medication. Nausea was categorized by using a 4-point Likert scale (0, no symptoms; 3, severe). CR and no nausea were measured in the acute phase (0-24 h), the delayed phase (24-120 h), and the overall phase (0-120 h). Secondary outcomes included the adverse events which was graded according to the common terminology criteria for adverse events (CTCAE) (5) and indicators related to QoL: Karnofsky performance scale (KPS) scores, weight, appetite, and sleep quality.

### 2.4 Quality Assessment

The quality of the included studies was assessed independently by two authors(SL, RD) based on the Cochrane Handbook for Systematic Review of Interventions (6). The Cochrane Collaboration’s tool for assessing the risk of bias for RCTs includes the following seven items: random sequence generation (selection bias), allocation concealment (selection bias), blinding of participants and personnel (performance bias), blinding of outcome assessments (detection bias), incomplete outcome data (attrition bias), selective outcome reporting (reporting bias), and other sources of bias. Each item was described as high risk of bias, low risk of bias, or unclear risk of bias. Disagreements were discussed and resolved by consensus between both reviewers or *via* consultation with a third reviewer (JX).

### 2.5 Statistical Analysis

Results were quantitatively synthesized by means of meta-analysis using the Review Manager (version 5.3; Cochrane Collaboration, Oxford, England). The Mantel-Haenszel method was used to estimate the pooled risk ratio (RR) for each dichotomous variable. I^2^ was used to evaluate heterogeneity across studies. When heterogeneity (I^2^≥50%) was detected, random-effects meta-analyses were performed. I^2^<50%, a fixed effect statistical model was used. Results obtained from the analyses were displayed by generating a forest plot. A p-value of < 0.05 was considered statistically significant.

## 3 Results

### 3.1 Study Selection and Trial Characteristics

**Figure 1:**
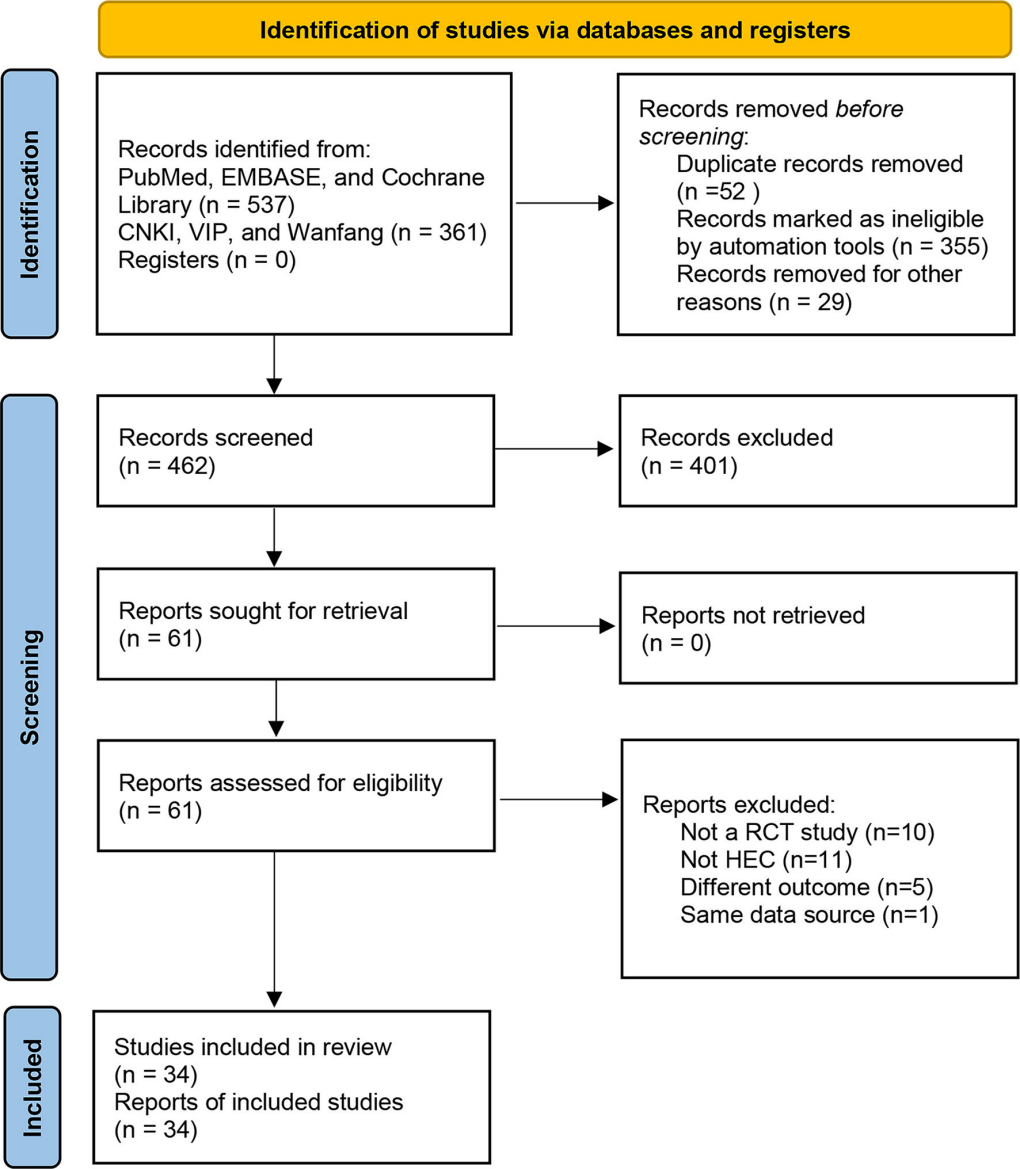
Flow diagram.

There were 898 records identified *via* database searching. 537 of the records were searched in PubMed, EMBASE, and the Cochrane library, 361 of the records were searched in the CNKI, VIP Information Database, and Wanfang Database (**Figure 1**).

After removing the duplicates, there were 462 results. The titles and abstracts of 462 studies were screened, and the full text of 61 articles was reviewed. 27 studies were excluded for the following reasons: not a RCT study (n = 10), not HEC (n = 11), a different outcome (n = 5), and same data source (n = 1). Finally, 34 studies were assessed for eligibility and included in the quantitative synthesis. A total of 3168 patients were included. The characteristics of the included studies are shown in **Table 1**. All studies were RCTs. Patients’ tumor types include breast, gastric, non-small cell lung cancer (NSCLC), small cell lung cancer (SCLC), cervical, and others. All patients in these studies received HEC.

**Table 1:** Characteristics of studies included.

Study	T/C(n)	Cancer Types	Chemotherapy Regimens	Interventions	Control	Outcomes
Cheng et al. (7)	45/45	Cervical	CDDP	THD(D0-4:50mg qn)+TRO+DEX	TRO+DEX	(2)(7)
Wang (8)	40/40	Lung	CDDP-containing	THD(100mg qn)+PAL+DEX	PAL+DEX	(3)
Song et al. (9)	40/43	Gastric/Lung/Cervical/Other	CDDP	THD(D1-5:100mg qd)+OND+MET+DEX	OND+MET+DEX	(1)(2)(3)(4)(5)(6)(7)
Zhang et al. (3)	317/321	Lung/Breast/Other	CDDP-containing/AC	THD(D2-4:100mg bid)+PAL+DEX	PAL+DEX	(1)(2)(3)(4)(5)(6)(7)
Li et al. (10)	30/30	Lung	CDDP-containing	THD(D1-5:100mg qn)+OND+DEX	OND+DEX	(1)(2)(4)(5)(7)
Zhao et al. (11)	39/39	Unknown	CDDP-containing/AC	THD(25mg bid)+TRO+DEX	TRO+DEX	(2)(7)
Han et al. (12)	40/38	Gastric/Lung/Ovarian	CDDP-containing	THD(D0:100mg qn,50mg was added per night up to 200 mg)+AZA	AZA	(2)(7)(8)
Han et al. (13)	38/32	Gastric/Lung/Ovarian	CDDP-containing	THD(D0:100mg qn,50mg was added per night up to 200 mg)+TRO	TRO	(3)(7)(8)
Zuo (14)	41/40	Breast	GP	THD(D1-8:25mg bid)+TRO	TRO	(1)(2)(4)(5)(7)
Cui et al. (15)	21/25	Breast	AC	THD(D1:25mg bid)+TRO	TRO	(1)(7)
Yu et al. (16)	30/31	NSCLC	GP	THD(D1-5:50mg bid)+RAM+MET	RAM+MET	(1)(2)(4)(5)(7)
Zhang et al. (17)	52/50	SCLC	CDDP-containing	THD(D1-7:100mg qn)+PAL+MP	TRO+MP	(1)(2)(4)(5)(7)(8)
Jiang (18)	138/128	Lung/Breast	CDDP-containing/AC	THD(D1-5:100mg bid)+PAL+DEX	PAL+DEX	(4)(5)(6)(7)
Xing et al. (19)	38/38	Gastric	DP	THD(D1-7:100mg qd)	Placebo	(7)
Luo (20)	26/28	NSCLC	GP	THD(D1-7:100mg qd D8-42: 200mg qd)	Placebo	(8)
Niu et al. (21)	32/28	Gastric	TP	THD(D1-42: 100mg qn)	Placebo	(7)(8)
Peng et al. (22)	51/53	NSCLC	TP	THD(D1-7:100mg qn D8-90:200mg qn)+5-HT_2_RA	5-HT_2_RA	(7)(8)
Peng et al. (23)	30/31	NSCLC	TP	THD(D1-7:100mg qn D8-90:200mg qn)+5-HT_2_RA	5-HT_2_RA	(7)(8)
He et al. (24)	19/20	NSCLC	NP	THD(D1-7:100mg qn D8-14:150mg qn D15-90: 200mg qn)+GRA	GRA	(7)
Zhang (25)	48/48	NSCLC	TP	THD(D1-7:100mg qd D8-84:200mg qd)+5-HT_2_RA	5-HT_2_RA	(7)
Gu et al. (26)	33/33	NSCLC	NP	THD(200mg qd)	Placebo	(7)(8)
Huang (27)	36/30	NSCLC	GP	THD(D1-30:200mg qn)+5-HT_2_RA	5-HT_2_RA	(8)
Pujol et al. (28)	49/43	SCLC	PCDE	THD(D1-112: 400mg qd)	Placebo	(7)
Sun and Xu (29)	30/30	NSCLC	DP	THD(D1-7:100mg qd D8-90:300mg qd)+GRA+DEX	GRA+DEX	(7)
Liang (30)	35/31	NSCLC	CDDP-containing	THD^a^	Placebo	(7)
Wang et al. (31)	60/60	NSCLC	GP	THD(D1-180:200mg qn)+5-HT_2_RA	5-HT_2_RA	(7)
Zuo (32)	37/37	SCLC	EP	THD(D6-21: 100mg/m^3^ 21d for 1 cycle, total 6 cycles of treatment)	Placebo	(7)
Xie et al. (33)	29/29	Breast	GP	THD(200mg qn)	Placebo	(7)
Dong (34)	30/30	NSCLC	TP	THD^b^+5-HT_2_RA	5-HT_2_RA	(8)
Huang and Wu (35)	30/30	NSCLC	TP	THD(D1-7:100mg qd D8-84:200mg qd)	Placebo	(8)
Liu et al. (36)	40/40	NSCLC	TP	THD(D1-7:100mg qd D8-90:200mg qd)+5-HT_2_RA	5-HT_2_RA	(8)
Jiang et al. (37)	31/30	NSCLC	GP	THD(D1-60:200mg qn)+AZA	AZA	(7)
Sun et al. (38)	36/21	NSCLC	NP	THD(D1-21:100mg bid)	Placebo	(7)
Shen et al. (39)	15/10	NSCLC	NP	THD^c^	Placebo	(7)

NSCLC, Non-small cell lung cancer; SCLC, Small cell lung cancer; CDDP, Cisplatin; AC, Anthracycline + Cyclophosphamide; GP, Gemcitabine+Cisplatin; DP, Docetaxel+Cisplatin; TP, Paclitaxel+Cisplatin; NP, Vinorelbine+Cisplatin; PCDE, Etoposide+Cisplatin+Cyclophosphamide+4-epidoxorubicin; EP, Etoposide+Cisplatin; THD, Thalidomide; TRO, Tropisetron; DEX, Dexamethasone; PAL, Palonosetron; OND, Ondansetron; MET, Metoclopramide; AZA, Azasetron; RAM, Ramosetron; MP, Methylprednisolone; GRA: Granisetron; 5-HT_2_RA: 5-HT_2_ receptor antagonist; (1): Complete response (acute phase); (2): Complete response (delayed phase); (3): Complete response (overall phase); (4): No nausea (acute phase); (5): No nausea (delayed phase); (6): No nausea (overall phase); (7): Adverse events; (8): Quality of Life.

a: 100mg qd (D1-7) and then weekly increase of 100 mg until reaching the tolerated dose.

b: 100mg qn (D1) and increase to 200 mg/d within one week, and then the maintenance dose lasts for 3 months.

c: 100mg qn (D1-7) and weekly increase of 50 mg until reaching the tolerated dose (400 mg/d is the maximum dose), treatment lasts for at least 3 months.

The included studies contained a total of 8 outcomes: CR (acute phase) (3, 9, 10, 14–17); CR (delayed phase) (3, 7, 9–12, 14, 16, 17); CR (overall phase) (3, 8, 9, 13); no nausea (acute phase) (3, 9, 10, 14, 16–18); no nausea (delayed phase) (3, 9, 10, 14, 16–18); no nausea (overall phase) (3, 9, 18); adverse events (3, 7, 9–19, 21–26, 28–33, 37–39); QoL (12, 13, 17, 20–23, 26, 27, 34–36).

### 3.2 Risk of Bias and Quality Assessment

**Figure 2:**
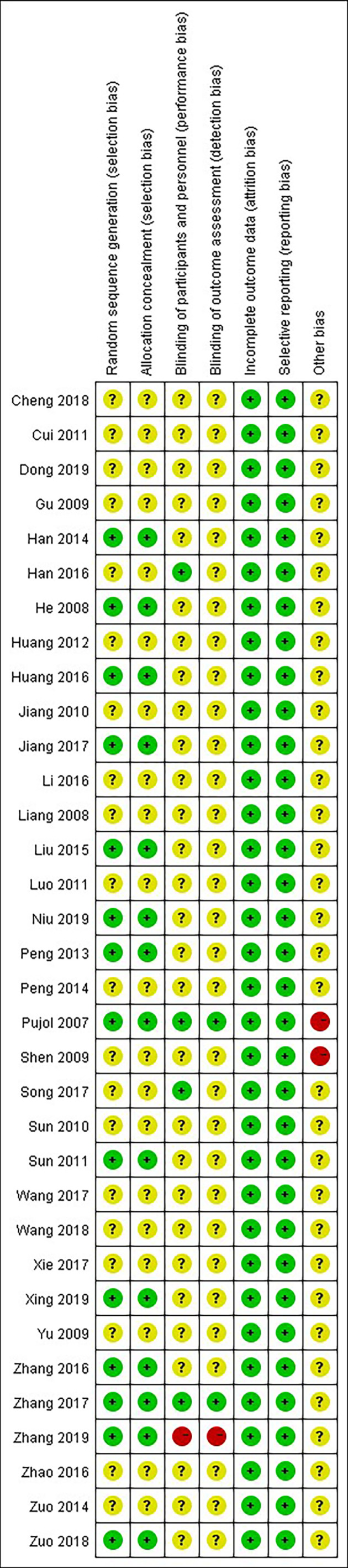
Assessment of risk of bias.

All of the included studies had a low risk of attrition bias and reporting bias. Only one study (25) had a high risk of performance bias and detection bias due to its single-blind method. Two of the included studies (28) and (39) had a high risk of other bias due to a possible conflict of interest or small sample size (**Figure 2**).

### 3.3 Primary Outcomes

#### 3.3.1 CR in the Acute Phase

**Figure 3:**
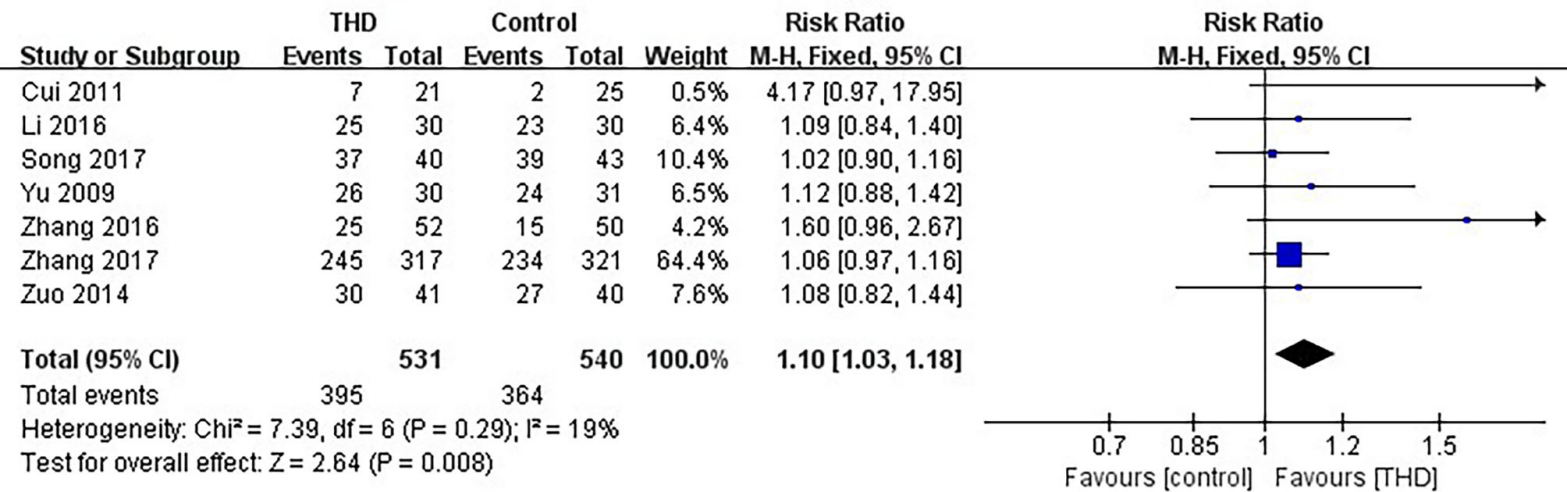
Meta-analysis on CR (acute phase).

Data of CR in the acute phase were available in 7 studies, including 1071 patients: 531 patients in the experimental group were treated with THD added to the 5-HT_3_RA-based conventional antiemetic regimen, and 540 patients in the control group were treated with the 5-HT_3_RA-based conventional antiemetic regimen. The CR rate was significantly higher with the addition of THD in the acute phase: 74.4% vs 67.4% (RR 1.10, 95%CI 1.03-1.18, p=0.008), without significant heterogeneity among studies (I²=19%) (**Figure 3**).

#### 3.3.2 CR in the Delayed Phase

**Figure 4:**
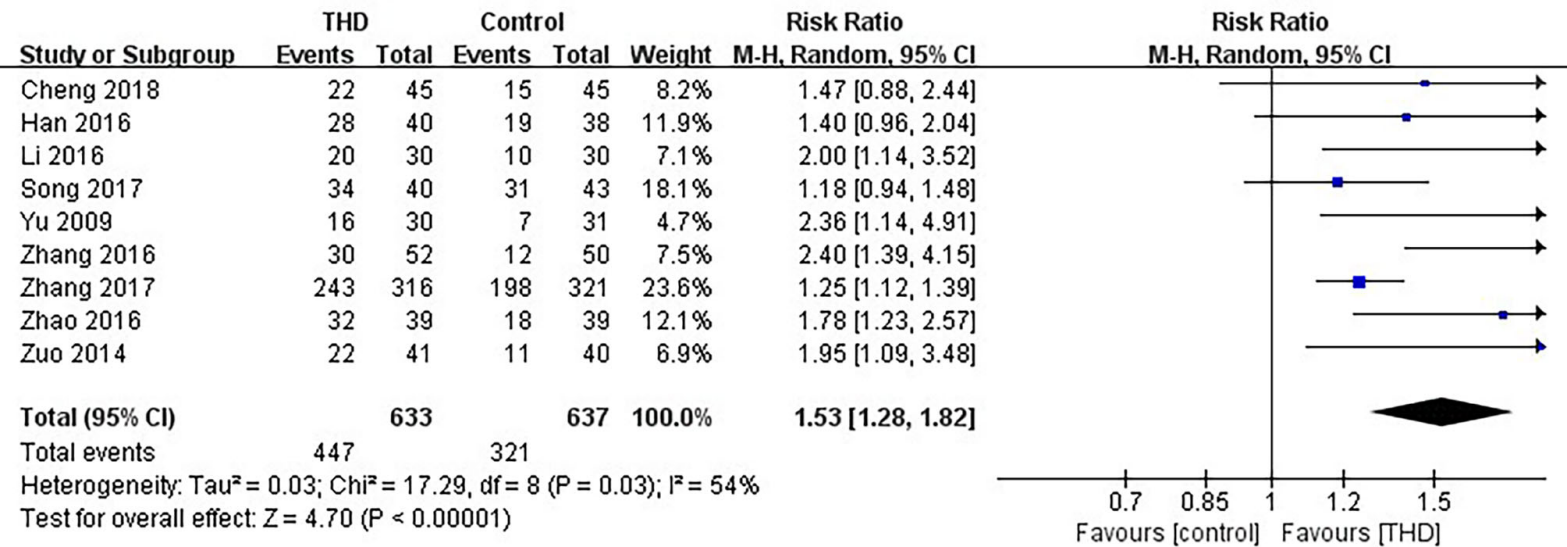
Meta-analysis on CR (delayed phase).

Data of CR in the delayed phase were available in 9 studies, including 1270 patients: 633 patients in the experimental group and 637 patients in the control group. The CR rate was significantly higher with the addition of THD in the delayed phase: 70.6% vs 50.4% (RR 1.53, 95%CI 1.28-1.82, p<0.00001), with significant heterogeneity among studies (I²=54%). Due to significant heterogeneity among the studies, a random-effects model was chosen for analysis (**Figure 4**).

#### 3.3.3 CR in the Overall Phase

**Figure 5:**
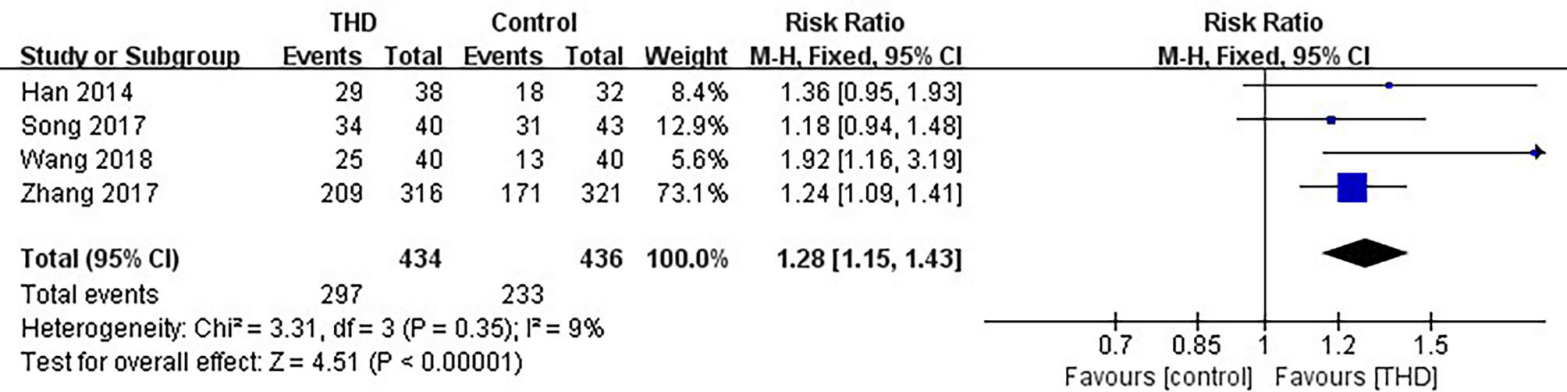
Meta-analysis on CR (overall phase).

Data of CR in the overall phase were available in 4 studies, including 870 patients: 434 patients in the experimental group and 436 patients in the control group. The CR rate was significantly higher with the addition of THD in the overall phase: 68.4% vs 53.4% (RR 1.28, 95%CI 1.15-1.43, p<0.00001), without significant heterogeneity among studies (I²=9%) (**Figure 5**).

#### 3.3.4 No Nausea in the Acute Phase

**Figure 6:**
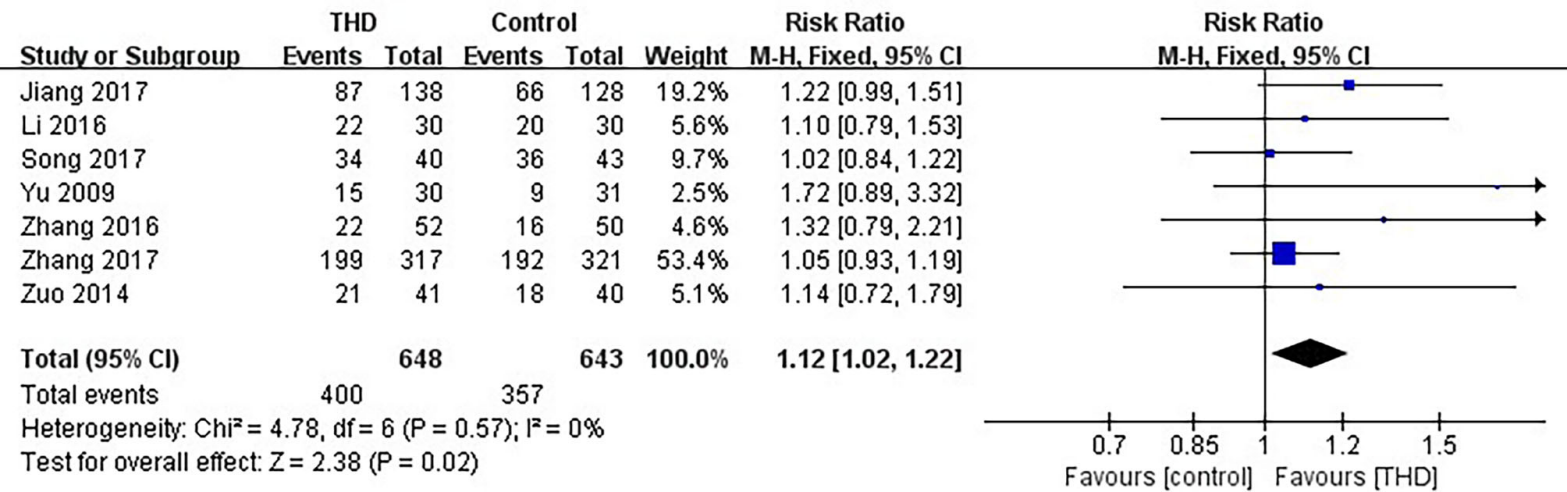
Meta-analysis on no nausea (acute phase).

Data of no nausea in the acute phase were available in 7 studies, including 1291 patients: 648 patients in the experimental group and 643 patients in the control group. The no nausea rate was significantly higher with the addition of THD in the acute phase: 61.7% vs 55.5% (RR 1.12, 95%CI 1.02-1.22, p=0.02), without significant heterogeneity among studies (I²=0%) (**Figure 6**).

#### 3.3.5 No Nausea in the Delayed Phase

**Figure 7:**
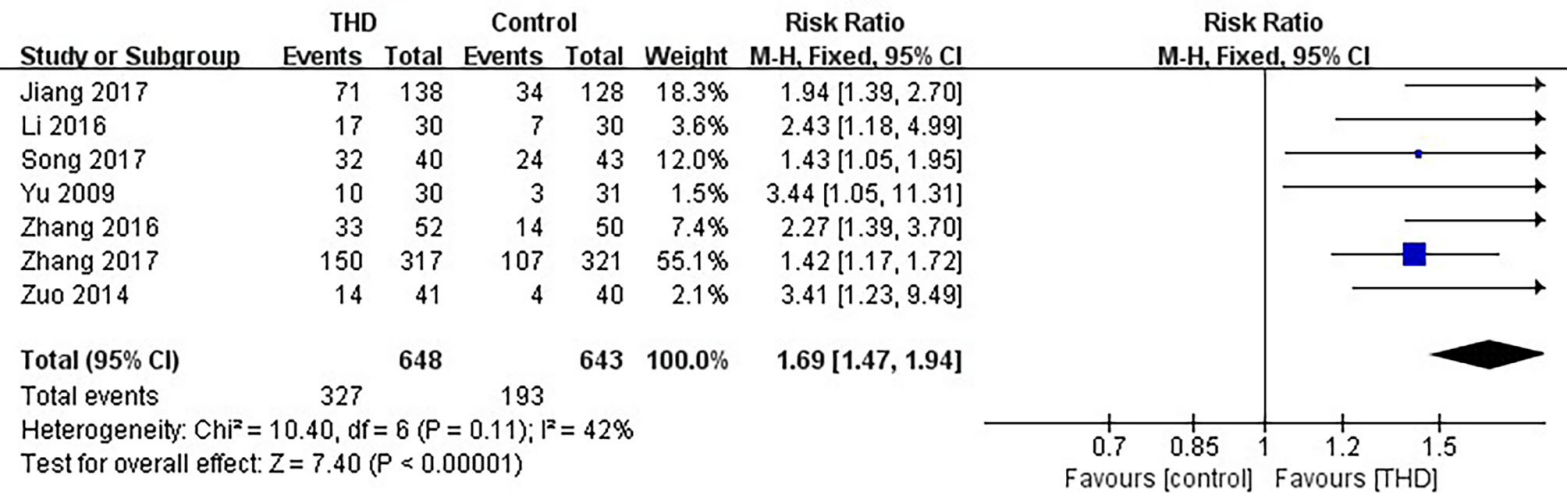
Meta-analysis on no nausea (delayed phase).

Data of no nausea in the delayed phase were available in 7 studies, including 1291 patients: 648 patients in the experimental group and 643 patients in the control group. The no nausea rate was significantly higher with the addition of THD in the delayed phase: 50.5% vs 30.0% (RR 1.69, 95%CI 1.47-1.94, p<0.00001), without significant heterogeneity among studies (I²=42%) (**Figure 7**).

#### 3.3.6 No Nausea in the Overall Phase

**Figure 8:**
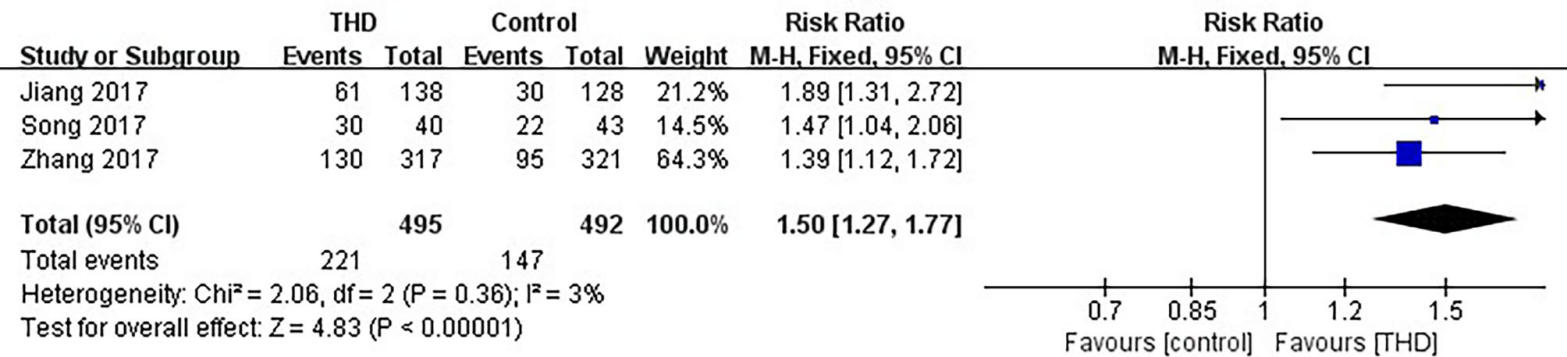
Meta-analysis on no nausea (overall phase).

Data of no nausea in the overall phase were available in 3 studies, including 987 patients: 495 patients in the experimental group and 492 patients in the control group. The no nausea rate was significantly higher with the addition of THD in the overall phase: 44.6% vs 29.9% (RR 1.50, 95%CI 1.27-1.77, p<0.00001), without significant heterogeneity among studies (I²=3%) (**Figure 8**).

### 3.4 Secondary Outcomes

#### 3.4.1 Adverse Events

Data from 28 out of the 34 included articles involved safety studies of THD and 11 adverse events were included: fatigue (12 studies), constipation (26 studies), mucositis (7 studies), headache (5 studies), diarrhea (7 studies), rash (11 studies), peripheral neuropathy (9 studies), hepatorenal damage (13 studies), myelosuppression (7 studies), somnolence (13 studies), and anorexia (3 studies). There was no significant heterogeneity among all studies (I²<50%) and all analyses were performed using a fixed-effects model.

The incidence of mucositis and anorexia was significantly lower with the addition of THD: namely, 14.6% vs 23.3% (RR 0.64, 95%CI 0.46-0.88, p=0.006) of mucositis; and 19.6% vs 37.4% (RR 0.52, 95%CI 0.34–0.81, p=0.003) of anorexia.

The incidence of constipation, peripheral neuropathy, and somnolence was significantly higher with the addition of THD: namely, 39.5% vs 26.9% (RR 1.45, 95%CI 1.30-1.61, p<0.00001) of constipation; 27.4% vs 16.2% (RR 1.61, 95%CI 1.25-2.08, p=0.0002) of peripheral neuropathy; and 25.9% vs 10.2% (RR 2.41, 95%CI 1.78-3.28, p<0.00001) of somnolence.

There was no statistical difference in the incidence of fatigue, headache, diarrhea, rash, hepatorenal damage, and myelosuppression between those with and without THD (p>0.05) (**Table 2**).

**Table 2:** Meta-analysis on adverse events.

Adverse Effects	Numberof trials	THD			Control			Heterogeneity analysis			Statistical analysis model	Statistical analysis	
		Events	Total	Incidence	Events	Total	Incidence	Chi²	P	I²		RR (95%CI)	P
Fatigue	12	333	837	39.8%	303	817	37.1%	19.53	0.05	44%	Fixed effect	1.06(0.95, 1.18)	0.3
Constipation	26	526	1333	39.5%	346	1285	26.9%	41.59	0.02	40%	Fixed effect	1.45(1.30, 1.61)	<0.00001
Mucositis	7	49	336	14.6%	72	309	23.3%	4.91	0.56	0%	Fixed effect	0.64(0.46, 0.88)	0.006
Headache	5	48	456	10.5%	52	454	11.5%	1	0.91	0%	Fixed effect	0.91(0.63, 1.31)	0.6
Diarrhea	7	54	640	8.4%	42	621	6.8%	5.47	0.49	0%	Fixed effect	1.22(0.84, 1.78)	0.3
Rash	11	55	484	11.4%	48	466	10.3%	12.24	0.27	18%	Fixed effect	1.09(0.76, 1.56)	0.64
Peripheral neuropathy	9	117	427	27.4%	63	388	16.2%	7.83	0.45	0%	Fixed effect	1.61(1.25, 2.08)	0.0002
Hepatorenal damage	13	59	474	12.4%	51	451	11.3%	4.94	0.96	0%	Fixed effect	1.06(0.76, 1.48)	0.72
Myelosuppression	7	86	260	33.1%	99	259	38.2%	7.24	0.3	17%	Fixed effect	0.88(0.71, 1.09)	0.25
Somnolence	13	121	468	25.9%	46	453	10.2%	23.72	0.02	49%	Fixed effect	2.41(1.78, 3.28)	<0.00001
Anorexia	3	22	112	19.6%	43	115	37.4%	3.84	0.15	48%	Fixed effect	0.52(0.34, 0.81)	0.003

#### 3.4.2 QoL

Data from 12 out of the 34 included articles examined the impact of THD on QoL and included 4 items: increase in the KPS scores (11 studies), weight gain (7 studies), appetite improvement (6 studies), and sleep quality improvement (4 studies). There was no significant heterogeneity among all studies (I²<50%) and all analyses were performed using a fixed-effects model.

The incidence of an increase in KPS scores, weight gain, appetite improvement, and sleep quality improvement was significantly higher with the addition of THD: namely, 55.9% vs 34.7% (RR 1.61, 95%CI 1.38-1.88, p<0.00001) of an increase in KPS; 49.4% vs 25.6% (RR 1.95, 95%CI 1.55-2.45, p<0.00001) of weight gain; 59.7% vs 41.0% (RR 1.47, 95%CI 1.23-1.74, p<0.00001) of appetite improvement; and 69.4% vs 25.9% (RR 2.66, 95%CI 1.92-3.69, p<0.00001) of sleep quality improvement (**Table 3**).

**Table 3:** Meta-analysis on QoL.

Quality of Life	Numberof trials	THD			Control			Heterogeneity analysis			Statistical analysis model	Statistical analysis	
		Events	Total	Incidence	Events	Total	Incidence	Chi²	P	I²		RR(95%CI)	P
Increase in KPS scores	11	227	406	55.9%	137	395	34.7%	6.42	0.78	0%	Fixed effect	1.61(1.38, 1.88)	<0.00001
Weight gain	7	131	265	49.4%	67	262	25.6%	5.35	0.5	0%	Fixed effect	1.95(1.55, 2.45)	<0.00001
Appetite improvement	6	139	233	59.7%	96	234	41.0%	1.04	0.96	0%	Fixed effect	1.47(1.23, 1.74)	<0.0001
Sleep quality improvement	4	86	124	69.4%	30	116	25.9%	4.53	0.21	34%	Fixed effect	2.66(1.92, 3.69)	<0.00001

## 4 Discussion

There is evidence that THD should be considered as an effective additional antiemetic medication (40). This meta-analysis suggests that the addition of THD to 5-HT_3_RA treatment (with or without DEX) is beneficial. Our findings showed that the addition of THD prevents CINV following HEC during the acute, delayed, and overall phase. Among these phases, the THD group had the most significant improvement in CINV during the delayed phase (70.6% vs 50.4% and 50.5% vs 30.0% in CR and no nausea, respectively).

This meta-analysis also suggests a high safety profile for the use of THD in patients with tumors undergoing HEC. Although the THD group increased the incidence of constipation, peripheral neuropathy, and somnolence, the incidence was significantly lower in mucositis and anorexia. The addition of THD did not increase the incidence of many adverse events (fatigue, headache, diarrhea, rash, hepatorenal damage, and myelosuppression). Researchers speculate that THD protects the oral mucosa by inhibiting NF-κB and supporting epithelial repopulation (41). Chemotherapy-induced intestinal mucositis and delayed diarrhea are associated with AIM2 (absent in melanoma 2) inflammasome activation, while THD eliminates AIM2 signaling and significantly reduces the incidence of drug-induced diarrhea (42). This study shows that there is no statistical difference in the incidence of diarrhea between the THD group and the control group, which may require more rigorous clinical trials and a wider population.

As a complementary drug, THD has been shown to improve QoL in cancer patients in this meta-analysis.

THD significantly improves KPS scores, weight, sleep quality, and appetite in cancer patients receiving HEC (55.9% vs 34.7%, 49.4% vs 25.6%, 59.7% vs 41.0%, and 69.4% vs 25.9%, respectively). A Cochrane meta-analysis shows that there is insufficient evidence to refute or support the use of THD for the treatment of cachexia in patients with advanced cancer (43). THD combined with megestrol acetate was shown to be effective in terms of appetite, body weight, and QoL (44).

This study has several strengths. Firstly, we included 34 RCTs and 3168 cases, expanding the scope and number of THD studies and greatly improving sample size and test efficacy. Secondly, we compared the differences in the incidence of 11 adverse events between the THD and control groups to provide a reference for the safety study of THD use in cancer patients. Finally, we also analyzed the effect of THD in increasing KPS scores, increasing weight, improving sleep quality, and increasing appetite from the perspective of QoL of cancer patients.

This meta-analysis also has some limitations. First, although the search for this study was extensive and included both English and Chinese databases, the final population of the literature included in the study was Chinese, which is not representative of other regional populations and ethnicities. Second, many of the studies we included scored poorly on quality assessment, which to some extent affects the final results of the meta-analysis. Finally, the number of studies containing the same outcome was no more than 10, so a funnel plot was not used to test for publication bias.

## 5 Conclusion

According to this systematic review and meta-analysis, we conclude that THD is effective and safe for the prevention of CINV in patients being treated with HEC, and has a significant tendency to improve QoL. More high-quality RCTs with more participants are warranted to support our findings.

## Data Availability Statement

The original contributions presented in the study are included in the article/supplementary material. Further inquiries can be directed to the corresponding authors.

## Author Contributions

JX and CZ contributed to study design, literature search, data collection, data analysis, and manuscript drafting. SL and RD contributed to quality assessment and data collection. MS, BD, and QX contributed to critical revision. JW, CS, and YZ contributed to conception, design, supervision, and manuscript drafting. All authors contributed to the article and approved the submitted version.

## Funding

This work was supported by the National Natural Science Foundation of China [82073402] and Key research and development program of Hubei Province [2020BCA060].

## Conflict of Interest

The authors declare that the research was conducted in the absence of any commercial or financial relationships that could be construed as a potential conflict of interest.

## Publisher’s Note

All claims expressed in this article are solely those of the authors and do not necessarily represent those of their affiliated organizations, or those of the publisher, the editors and the reviewers. Any product that may be evaluated in this article, or claim that may be made by its manufacturer, is not guaranteed or endorsed by the publisher.
